# Genome-Wide Identification of MicroRNAs in Response to Cadmium Stress in Oilseed Rape (*Brassica napus* L.) Using High-Throughput Sequencing

**DOI:** 10.3390/ijms19051431

**Published:** 2018-05-10

**Authors:** Hongju Jian, Bo Yang, Aoxiang Zhang, Jinqi Ma, Yiran Ding, Zhiyou Chen, Jiana Li, Xinfu Xu, Liezhao Liu

**Affiliations:** College of Agronomy and Biotechnology, Chongqing Engineering Research Center for Rapeseed, Academy of Agricultural Sciences, Southwest University, Beibei, Chongqing 400715, China; jianhongju1989@126.com (H.J.); sheepneck@hotmail.com (B.Y.); 13527593223@126.com (A.Z.); jinqima@outlook.com (J.M.); yrding1226@163.com (Y.D.); 15213269227@163.com (Z.C.); ljn1950@swu.edu.cn (J.L.); xinfuxu@126.com (X.X.)

**Keywords:** miRNAs, cadmium stress, gene regulation, *Brassica napus*

## Abstract

MicroRNAs (miRNAs) have important roles in regulating stress-response genes in plants. However, identification of miRNAs and the corresponding target genes that are induced in response to cadmium (Cd) stress in *Brassica napus* remains limited. In the current study, we sequenced three small-RNA libraries from *B. napus* after 0 days, 1 days, and 3 days of Cd treatment. In total, 44 known miRNAs (belonging to 27 families) and 103 novel miRNAs were identified. A comprehensive analysis of miRNA expression profiles found 39 differentially expressed miRNAs between control and Cd-treated plants; 13 differentially expressed miRNAs were confirmed by qRT-PCR. Characterization of the corresponding target genes indicated functions in processes including transcription factor regulation, biotic stress response, ion homeostasis, and secondary metabolism. Furthermore, we propose a hypothetical model of the Cd-response mechanism in *B. napus*. Combined with qRT-PCR confirmation, our data suggested that miRNAs were involved in the regulations of TFs, biotic stress defense, ion homeostasis and secondary metabolism synthesis to respond Cd stress in *B. napus*.

## 1. Introduction

Increasing heavy metal pollution in aquatic and terrestrial environments, caused in part from the burning of fossil fuels and mining, has drawn worldwide attention [1]. Of the seven key heavy metal pollutants, cadmium (Cd) is one of the most harmful to both ecosystems and human health [2,3]. Although Cd ions (Cd^2+^) are not essential for plants, they are taken in easily by roots and then transported to the above-ground organs [4]. Excess Cd accumulation can cause detrimental effects on plant growth and development, including inhibition of photosynthetic activity [5], membrane distortion [6], stunted plant growth [7,8], decreased nutrient uptake [9], and decreased crop yield [4,10]. To tolerate Cd stress, plants have evolved elaborate protective mechanisms and extensive research has begun to reveal these mechanisms [11,12]. For example, AtPDR8 (PLEIOTROPIC DRUG RESISTANCE 8, an ABC transporter) acts as a Cd^2+^ efflux pump in the plasma membrane of Arabidopsis cells [13]. The Zn/Cd transporters HMA4 (P-type ATPase) in *Arabidopsis halleri* and HMA3 (ATPase) in *Thlaspi caerulescens* were reported to be involved in Cd regulation [14]. In tobacco (*Nicotiana* spp.), over-expression of *OsACA6* resulted in improved Cd^2+^ stress tolerance by maintaining cellular ion homeostasis and regulating the reactive oxygen species (ROS)-scavenging pathway [15]. In addition, heavy metal stress signals induced through various pathways have been identified; these include mitogen-activated protein kinases (MAPKs) [16], hormones (e.g., salicylic acid) [17], Ca-calmodulin system [18], ROS signaling [19], and corresponding transcription factors (TFs) [20,21].

Though studies of the mechanisms on physiological and biochemical levels have been conducted [22], the regulatory mechanisms in response to Cd^2+^ stress on genetics, transcriptional and post-transcriptional levels are still vague. To explore the mechanisms regulating the response to Cd^2+^ stress, scientists have identified Cd-responsive genes and characterized their regulatory networks. Cd-responsive TFs have been identified in Arabidopsis [23], pea (*Pisum* spp.) [19] and barley (*Hordeum* spp.) [24], including WRKY53 [25], basic leucine zipper (bZIP) [26], myeloblastosis protein 4, 48 and 124 (MYB4, 48 and 124) [20,23], and ethylene-responsive factor 1/2 (ERF1/2) [23]. These Cd-responsive TFs are essential for regulating the expression of genes associated with the Cd stress response.

Numerous studies have demonstrated the importance of miRNAs in many biological processes including organ development, signal transduction, as well as biotic and abiotic stress responses at post-transcriptional levels. Moreover, miRNAs were identified as key roles in heavy metal regulatory networks. For example, nineteen Cd-responsive miRNAs in rice (*Oryza* spp.) have been identified using microarrays [27]. Furthermore, 39 known and 8 novel miRNAs were differentially expressed in response to Cd treatment in rice [28]. A recent study with radish (*Raphanus* spp.) identified 23 Cd-responsive miRNAs and characterized the corresponding targets associated with metal transport and signaling [29]. In *Typha angustifolia*, 155 miRNAs (114 conserved and 41 novel miRNAs) were screened after Cd exposure [1]. All of these evidences strongly support the key roles of miRNAs in heavy metal stress responses at the post-transcriptional level.

Oilseed rape (*Brassica napus* L.) is a globally important economic crop due to its seed oil, which is used for food and biofuel. Several studies have been conducted to understand the physiological responses of *B. napus* to Cd stress [22,30,31,32,33]. Recently, a genome-wide identification of miRNAs involved in agronomic traits, abiotic stress, biotic stress, and oil content in *B. napus* was performed [34,35,36,37]. Furthermore, several Cd^2+^ stress responsive miRNAs have been reported and related regulatory mechanisms have also been discussed [38,39,40]. Noteably, miR395 and corresponding targets (*BnSultr2;1*, *BnAPS3* and *BnAPS4*) were identified as key roles in the heavy metal stress [39]. In another study, 802 target genes for 37 miRNA families were also screened as regulators in response to Cd stress [40]. Even though many miRNAs in *B. napus* have been revealed using high-throughput sequencing technology, heavy metal-regulated miRNAs (especially those related to Cd^2+^ stress) and corresponding target genes have not been entirely identified. Thus, identifying and characterizing the miRNAs involved in a Cd^2+^ stress response in *B. napus* may be useful for understanding the mechanisms of heavy metal tolerance.

In the present study, genome-wide identification of miRNAs in response to Cd stress was conducted in *B. napus* using high-throughput sequencing technology. In total, 44 known and 103 novel miRNAs were identified and 39 miRNAs were differentially expressed. Additionally, an integrated schematic diagram was created based on differentially expressed miRNAs and corresponding target genes to better understand the regulation mechanisms related to Cd stress tolerance. These results extend our knowledge of miRNA-guided regulation of heavy metal stress responses in *B. napus*.

## 2. Results

### 2.1. High-Throughput Sequencing of Small RNAs

**Figure 1:**
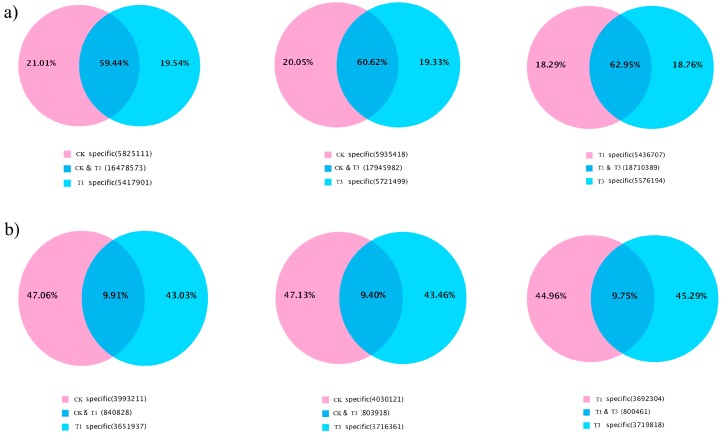
Venn diagrams for analysis of total (**a**) and unique (**b**) reads between each of the two samples of *B. napus.*

**Figure 2:**
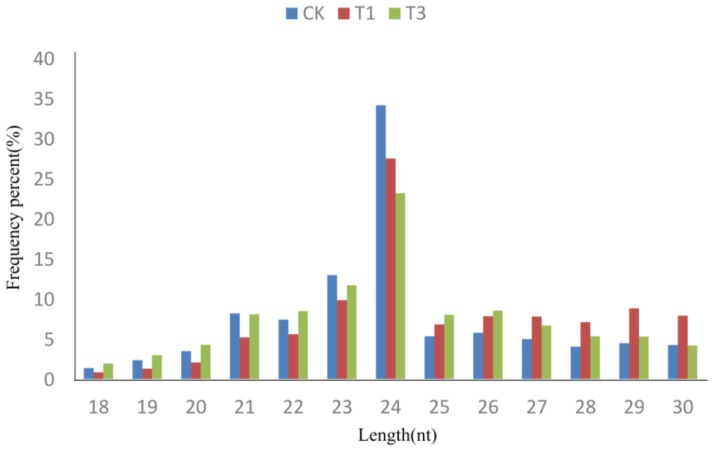
Length distribution of small RNAs in three samples. Y-axis: the percentage of small RNA reads.

Three sRNA libraries were generated and sequenced using Illumina sequencing technology. High-throughput sequencing generated 16,892,251 reads in the CK library, 20,019,156 reads in the T01 library, and 19,891,839 reads in the T03 library. After removing adaptors, contamination and sequence lengths greater than 30 or less than 18, a total of 13,800,597 clean reads were obtained for CK, 13,920,988 for T01, and 15,802,302 for T03 (Table 1). The proportions of common and specific small RNAs were further analyzed between pairs of libraries. For total small RNAs, 59.44, 60.62% and 62.95% were common detected in CK and T1, CK and T3, and T1 and T3, respectively; 18.29–21.01% were specific detected in CK, T1 and T3 library (Figure 1a). However, for unique small RNAs, 9.40–9.91% were common detected in CK and T1, CK and T3, and T1 and T3 comparisons and 43.03–47.31% were specific detected in three samples (Figure 1b). The size distribution of all sRNAs (18–30 nt) was uneven, the 24-nt classes showed the highest degree of redundancy in all three libraries (Figure 2).

### 2.2. Identification of Known and Novel miRNAs in B. napus

In general, miRNAs are highly conserved among species. The miRBase database (v21.0, http://www.mirbase.org/index.shtml) [41,42], which contains 92 mature miRNAs for *B. napus*, was used to detect known miRNAs with no more than two mismatches in the three libraries generated for the current work. A total of 44 known *B. napus* miRNAs, belonging to 27 families, were identified in this study (Table 2). Of these families, 18 families were identified containing one miRNA, 6 families containing more than three miRNAs, and 3 families containing two miRNAs (miR164, miR166 and miR395) (Table 2). In addition, 35 out of 44 miRNAs had a length of 21 nt, whereas 3, 5 and 1 miRNAs had lengths of 20 nt, 23 nt and 24 nt, respectively (Table 2). Furthermore, miR169g and miR211c were CK-specific, miR399a was T01-specific, and miR2111a-5p was T03-specific. All other miRNAs were commonly detected in the three libraries (Table 2).

**Figure 3:**
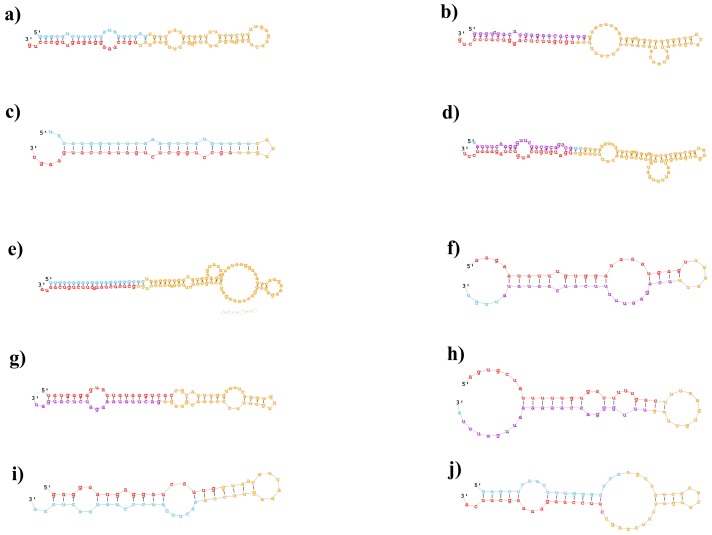
Predicted secondary structures of selected eight novel miRNAs in our study. The red indicates mature miRNA sequences, yellow indicates ring structure and purple indicates star sequences. (**a**) Novel-004; (**b**) Novel-036; (**c**) Novel-013; (**d**) Novel-018; (**e**) Novel-049; (**f**) Novel-059; (**g**) Novel-073; (**h**) Novel-092; (**i**) Novel-010; (**j**) Novel-015.

To identify novel miRNAs in *B. napus*, RNAfold and Mireap were utilized. A characteristic stem-loop precursor is the most important characterization for new miRNAs (Meyers et al., 2008). In the current study, a total of 103 small RNAs were regarded as candidate novel miRNAs in *B. napus* (Table 3). These novel miRNAs showed a length distribution between 18 nt and 25 nt (but no miRNAs were 19 nt in length) with 44 miRNAs of 21 nt long and 32 miRNAs of 24 nt long. (Table 3). Similar to past analyses with *B. napus* and other plant species, the minimum free energy (AMFE) varied from −171.9 to −26.9 kcal moL^−1^ (Table 3). The precursor length of these miRNAs was 250 nt, and some selected secondary structures are shown in Figure 3.

### 2.3. Target Prediction and Functional Analysis of miRNAs

Target prediction is crucial to understanding the functions of miRNAs. In this study, 460 putative target genes were predicted for 33 known and 44 novel miRNAs with an average of 5.97 targets per miRNA (psRNA Target, 2011 release; http://plantgrn.noble.org/psRNATarget/) [43]. Of the 77 miRNAs, miR156d had the most putative targets (40) among the known miRNAs, and Novel-015 had the most potential targets (50) within the novel miRNAs (Tables S1 and S2).

**Figure 4:**
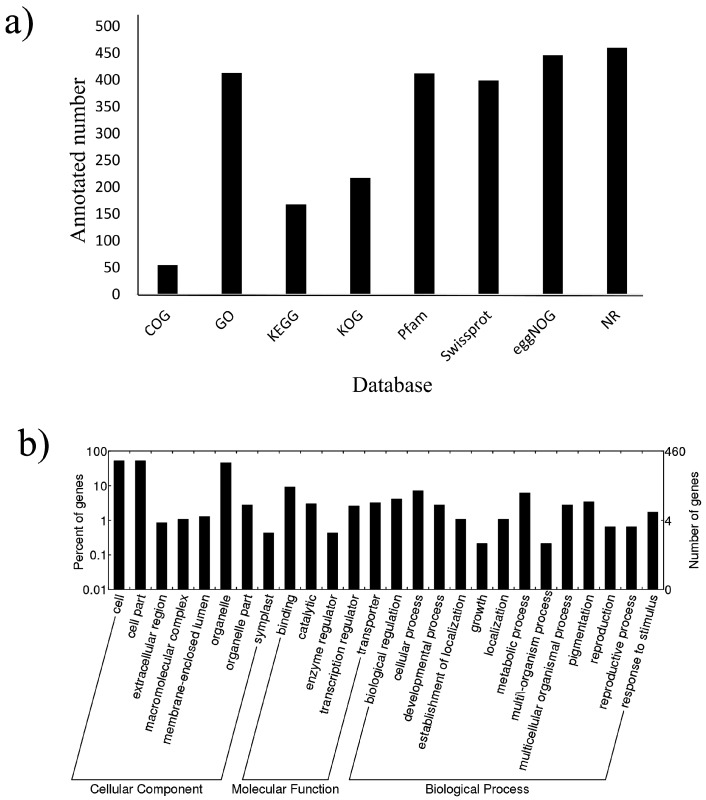
Diagrams of differentially expressed known (**a**) and novel (**b**) miRNAs between each treatment comparison.

To further understand the crucial roles of miRNAs, all target genes were annotated in eight databases. Only 55 genes were annotated in the COG database, and almost all genes were annotated in the Swissprot (399), Pfam (412), GO (413), eggnog (446), and NR (460) databases (Figure 4a). In addition, all target genes were sent to GO functional classification (http://www.geneontology.org/) [44]. The results showed that these target genes were involved in 26 terms related to 8 cellular components (cell/cell part), 5 molecular functions (binding process) and 13 biological processes (metabolic process) (Figure 4b). Interestingly, eight target genes were involved in stimulus response processes (GO: 0050896).

### 2.4. qRT-PCR Validation

**Figure 5:**
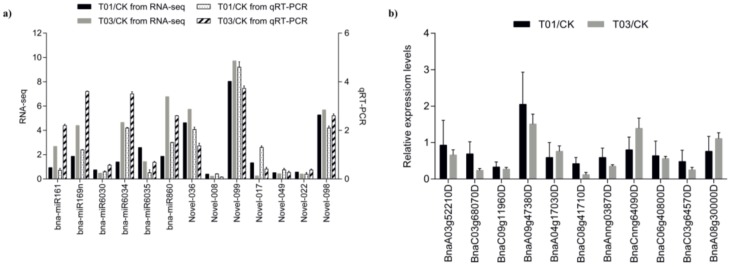
Validation and comparison of the expression of 13 miRNAs between qRT–PCR and small RNA sequencing (**a**) and 11 corresponding target genes (**b**). Fold change of miRNA expression relative to the control sample.

To test the reliability of high-throughput sequencing results, the expression patterns of 13 miRNAs (including 6 known and 7 novel miRNAs) were observed using qRT-PCR. As expected, the qRT-PCR demonstrated a high consistency with sRNA sequencing data (Figure 5a). The correlation coefficient between NGS and qRT-PCR data were 0.7079 (*P* < 0.05) and 0.7694 (*P* < 0.05) using T1/CK and T3/CK, respectively, suggesting the NGS were reliable. The sensitivity of the fold changes between the two different methodologies were slightly different among three samples. Eleven target genes including four for miR6030, four for Novel-010, two for Novel-015 and one for Novel-090 were used to detect expression changes (Figure 5b). Reverse expression changes with miRNAs were detected indicating these predicted target genes were possibly regulated by corresponding miRNAs except these pairs: Novel-015 and *BnaA03g52210D*, miRNA Novel-10 and *BnaA08g30000D*, miR3060 and *BnaC08g41710D* and *BnaAnng03870D*. The possible reasons are: (1) these genes we selected may not real targets of corresponding miRNAs; (2) the low expression level of miRNAs or (3) these targets were not degrading immediately.

### 2.5. miRNA Expression in Response to Cd Stress

**Figure 6:**
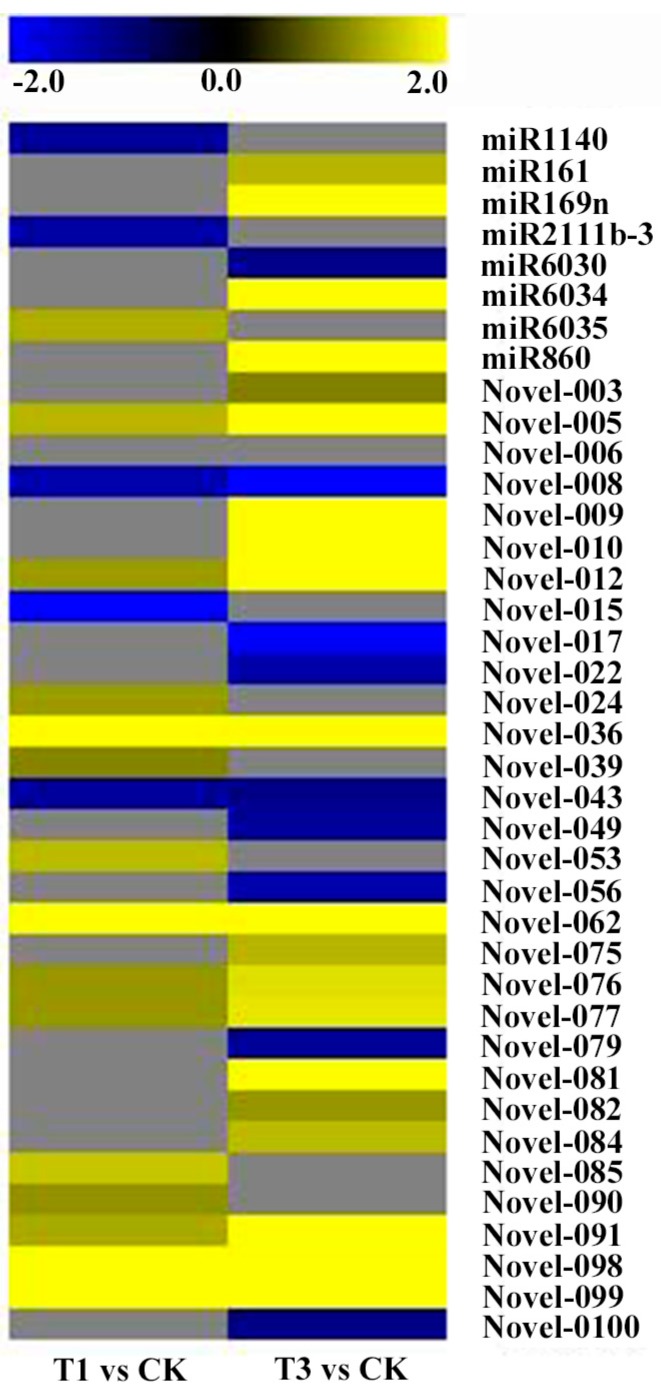
Differential expression of conserved miRNAs and novel miRNAs between two samples.

**Figure 7:**
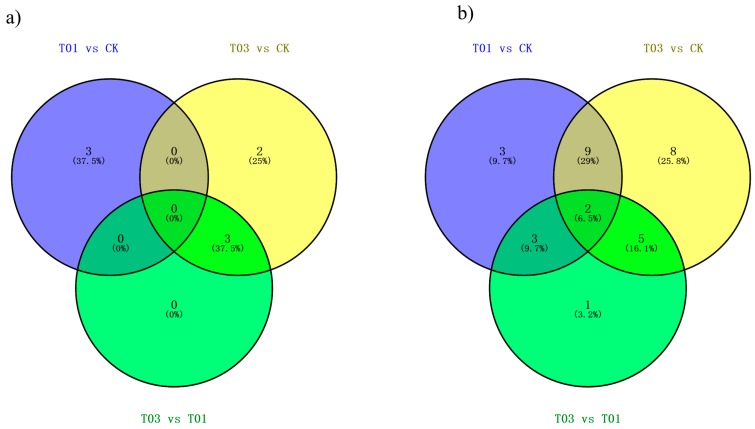
Annotation of predicted target genes for miRNAs. (**a**) Number of target genes in various databases; (**b**) Histogram of gene ontology (GO) classification of predicted target genes. The left and right y-axis indicates the percentage and number of a specific category of genes in that main category, respectively.

Among the 44 known miRNAs, 3 miRNAs were differentially expressed in T01 comparing CK library (miR1140 and miR2111b-3p were down-regulated while miR6035 was up-regulated). When comparing T03 to CK, 5 miRNAs were differentially expressed (miR161, miR169n, miR6034 and miR860 were up-regulated while miR6030 was down-regulated) (Figure 6). In addition, miR161, miR169n and miR6034 were differentially expressed in comparisons of T03 vs CK and T03 vs T01. Lastly, 3, 2 and 0 miRNAs were specific differentially expressed in comparison of T01 vs. CK, T03 vs. CK, and T03 vs. T01, respectively (Figure 7a).

From the novel 103 miRNAs, 31 were differentially expressed among the three libraries (Figure 6). When comparing T01 to CK, 17 were differentially expressed (3 down-regulated and 14 up-regulated). A comparison of T03 to CK demonstrated 24 that were differentially expressed (8 down-regulated and 16 up-regulated). Lastly, when comparing between T03 and T01, there were 11 differentially expressed (5 down-regulated and 6 up-regulated) (Figure 6). Furthermore, 3 miRNAs were specific differentially expressed between T01 and CK, 8 miRNAs between T03 and CK, and 1 miRNA between T03 vs T01. The two miRNAs Novel-062 and Novel-012 were common differentially expressed in all three comparisons (Figure 7b).

## 3. Discussion

**Figure 8:**
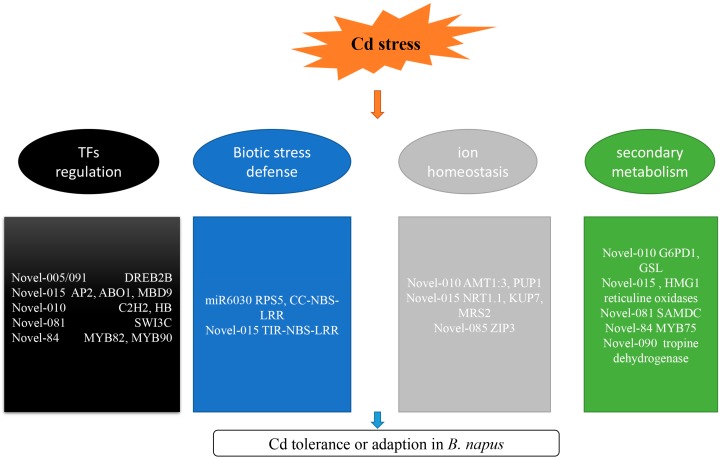
A hypothetical integrated schematic diagram of the mechanisms involved in Cd tolerance in *Brassica napus*.

Cadmium is a widespread and toxic heavy metal pollutant that is detrimental to food and drinking water supplies [45]. *B. napus*, one of the most important oil and biofuel crops, is also under threat even though it is moderately tolerant of heavy metals [40]. To adapt to cadmium stress, fine and complex regulatory mechanisms have evolved in plants. In the last few years, miRNAs have been regarded as crucial regulators in a plant’s response to toxic metal stress [46]. Identification of miRNAs and corresponding target genes is necessary when exploring regulatory stress mechanisms. In the current study, miRNA profiles were generated of *B. napus* seedlings exposed to Cd stress. In total, 39 differentially expressed miRNAs were identified, of which 8 are known and 31 are novel. Cd responsive miRNAs may contribute to Cd tolerance by regulating transcription factors (TFs) that are important in biotic stress responses, ion transporters and secondary metabolism (Table S3, Figure 8).

### 3.1. miRNAs Involved in TF Regulation

TFs have central roles in plant growth and development, as well as biotic and abiotic stresses. Several TFs that are responsive to heavy metal stress have been identified previously [21]. APETALA2 (AP2)/ethylene-responsive-element-binding protein (EREBP) family and MYeloBlastosis Protein (MYB) proteins are involved in essential responses to heavy metal stress. Additionally, several members of AP2 TF can regulate pathogenesis-related and dehydration responsive genes by binding their promoters [47]. In a previous work, researchers have suggested that *ERF1* and *ERF2* genes were involved in a response to Cd treatment in *Arabidopsis thaliana* roots [23]. In addition, *DREB2A* was also induced by Cd. In the current study, five *AP2* genes were predicted as target genes for novel-05, novel-15 and novel-91. *BnaAnng23490D* (*DREB2B*) was a common target for novel-05 and novel-91, which were both up-regulated after 1 and 3 days of Cd treatment. However, novel-15 was down-regulated after 1 day of Cd treatment. In *A. thaliana*, *MYB4* was induced after Cd and Zn-treatments [20], while *MYB43*, *MYB48* and *MYB124* were strongly and specifically expressed after Cd treatment in roots [23]. Glucosinolate (GSL) has important functions in the response to nutritional status, and biotic and abiotic stresses [48]. Its synthesis is regulated by *MYB28*, which is highly induced by Zn deficiency and exposure to high concentrations of Cd [20]. Currently, three *BnMYB*s (two *BnMYB82*s and one *BnMYB90*) genes were targeted by novel-84, which was up-regulated after 3 days of Cd treatment. Moreover, some other TFs such as C_2_H_2_, HB and ABO1 (ABA-OVERLY SENSITIVE 1) were also detected as target genes for differentially expressed miRNAs.

### 3.2. miRNAs Involved in Biotic Stress Responses

Previous researches identified crosstalk between pathogen defense signaling and Cd regulation mechanisms [26]. *RESISTANT TO P. SYRINGAE 5* (*RPS5*) is involved in resistance against the bacterial pathogen *Pseudomonas syringae* [49]. Five *RPS5* genes and 11 disease resistance protein genes (CC-NBS-LRR class) were targeted by miR6030, which was down-regulated after 3 days of Cd treatment. In addition, three PR-protein genes (TIR-NBS-LRR class) were predicted as target genes for novel-15, which was down-regulated after 1 day of Cd treatment. Moreover, another two genes *BnaA01g20350D* and *BnaA07g17000D*, involved in biotic stress were also predicted as target genes for novel-10 and novel-15, respectively. These data suggest that stresses associated with heavy metals and biotic factors may share the same signal transduction pathway.

### 3.3. miRNAs Involved in Ion Transport

In general, crops take up metal ions and nutrients from soil using highly effective transmembrane protein transporters. In this system, metal transporters located in the plasma membrane or tonoplast, have crucial roles in ion homeostasis. If a toxic Cd ion was drawn into a cell, the ion could be transported out or sequestered to the vacuole. In Arabidopsis, the important metal transporter ZIP (ZRT, IRT-like protein) family, located in plasma membranes, was induced in both shoots and roots in response to Zn-limiting conditions. ZIP proteins have essential roles in divalent cation transport for many plant species [50]. Several members of the ZIP family function in Cd uptake and transport, and are involved in the xylem unloading process [51]. In the current study, *BnaC04g42780D* (*ZIP3*) was predicted as a target gene for novel-85, which was up-regulated by 1.5 fold after only 1 day of Cd treatment. Cadmium can also influence water uptake and nutrient metabolism [52]. In total, four magnesium transporter genes (*MRS2*), one potassium ion transmembrane transporter gene (*KUP7*) and one nitrate transmembrane transporter gene (*NRT1.1*) were predicted as target genes for novel-015, which was down-regulated after 1 day of Cd treatment. One purine nucleoside transmembrane transporter gene (*PUP1*) and one ammonium transmembrane transporter gene (*AMT1:3*) were targeted by novel-010, which was up-regulated after 3 days of Cd treatment. Other transporters such as NRAMP (natural resistance-associated macrophage protein) [53], HMA (P_1_B-ATPases) [51], ABC transporters [54] and CDF (cation diffusion facilitator) [51] are crucial for metal transport and ion homeostasis in Arabidopsis. However, no homologs of these genes were predicted as target genes for differentially expressed miRNAs in *B. napus*. Some reasons for this include differences in Cd concentration, duration of Cd treatment and sequencing depth.

### 3.4. miRNAs Involved in Secondary Metabolism

Secondary metabolites have important functions in response to biotic and abiotic stresses [55]. Genes involved in the biosynthesis of flavonoids and isoprenoids were predicted as target genes for miRNAs in this study. Flavonoids were used in transgenic engineering to enhance resistance to various stresses [56]. PRODUCTION OF ANTHOCYANIN PIGMENT 1 (PAP1) contains a MYB domain and is involved in anthocyanin metabolism and radical scavenging [57]. In *myb12* mutants, PAP1 transcription did not occur in response to auxin and ethylene [58]. PAP1 regulates anthocyanin accumulation by interacting with JAZ proteins [59]. Currently, five homologous *PAP1* genes in *B. napus* were predicted as target genes for novel-084, which was up-regulated after 3 days of Cd treatment. *HYDROXY METHYLGLUTARYL COA REDUCTASE 1* (*HMG1*) encodes a 3-hydroxy-3-methylglutaryl coenzyme A reductase, and is involved in the first step in isoprenoid biosynthesis [60]. Its expression is activated in leaf tissue under dark conditions but not regulated by light in roots. Two copies of HMG1 in *B. napus* were predicted as target genes for novel-015, which was down-regulated after 1 day of Cd treatment. Other genes involved in secondary metabolite synthesis were also predicted as target genes for differentially expressed miRNAs.

Based on the microRNA profile analysis with or without Cd treatment, 4 known miRNAs and 12 novel miRNAs were identified as being responsive to Cd tolerance in *B. napus*. These miRNAs were mainly involved in TFs, biotic stress defense, ion homeostasis and secondary metabolism synthesis. The results provide more information about the mechanism of toxic metal resistance in *B. napus* and other important crops.

## 4. Materials and Methods

### 4.1. Plant Culture and Treatment

Seeds of *Brassica napus* (Zhongshaung 11) were surface sterilized in 1.2% NaOCl and germinated at 22 ± 1 °C for 7 d on a plastic net floating on distilled water. Seedlings were then transferred to 1/4-strength modified Hoagland nutrient solution for another 7 d with a 14/10 h light/dark cycle at 22 ± 1 °C and 250 μmoL m^−2^ s^−1^ light intensity. Next, the seedlings were transferred to fresh nutrient solution containing either 0 μM or 1000 μM CdCl_2_ for 0 d, 1 d, or 3 d. Following this treatment, seedlings (including roots and shoots) were harvested, frozen immediately in liquid nitrogen, and stored at −80 °C.

### 4.2. Construction and Sequencing of Small RNA Libraries

Small RNA libraries were created based on our previous work [34]. Total RNA was isolated from whole *B. napus* plants using Trizol (Invitrogen, Carlsbad, CA, USA) according to the manufacturer’s instructions. Three sets of total RNA were prepared from samples after 0 d (CK), 1 d (T01) and 3 d (T03) treatments. The quality of the RNA samples was checked with an Agilent 2100 Bioanalyzer (Agilent, Santa Clara, CA, USA) and then all three RNA samples were sent to Beijing Biomarker Technologies Co., Ltd. (Beijing, China) for sRNA library construction and Solexa sequencing using the Illumina HiSeq 2500 platform. Furthermore, all sequencing data were uploaded on NCBI with accession number SRP118762.

### 4.3. Analysis of Small RNA Sequencing Data

Clean data were screened from raw data by removing contaminants, adaptors, and low-quality reads. Unique reads were then mapped on a *B. napus* reference genome (http://www.genoscope.cns.fr/brassicanapus/) [61] using the SOAP2 program with default parameters [62]. Sequences with a best match were retained and those that were matched on non-coding RNAs, exclusive miRNAs in the Rfam 13.0 (http://www.sanger.ac.uk/Software/Rfam), and NCBI GenBank 225.0 (http://www.ncbi.nih.gov/GenBank/) databases were removed. The website miRBase 21.0 (http://www.mirbase.org/index.shtml) was used to identify known miRNAs with a maximum of two mismatches. The remaining unmapped sequences were used to predict novel miRNAs using Mireap_0.2 software (https://sourceforge.net/projects/mireap/) with basic criteria [63].

### 4.4. Differential Expression Analysis of miRNAs under Cd Stress

To screen Cd-responsive miRNAs, their expression (including known and novel miRNAs) in each sample was normalized using following formula:

normalized expression = actual miRNA count/total count of clean reads × 1,000,000

The expression value was regarded as 0.01 for further analysis if the read count of a miRNA was 0. The fold change between the T01 or T03 and control (CK) sample was calculated as:

fold change = log_2_ (T01 or T03/CK)

The miRNAs with absolute value of fold changes ≥1 and with *P* ≤ 0.05 were considered to be Cd-responsive miRNAs. FDR (False positive rate) was calculated using the Benjamin correction value of the statistical *P* value and the software IDGE6 was used to select differentially expressed miRNAs.

### 4.5. Prediction of miRNA Targets

The website psRNATarget 2011 (http://plantgrn.noble.org/psRNATarget/) was used to predict target genes of miRNAs according to default parameters. Moreover, gene ontology (GO) was used for function annotation of the target genes in Blast2GO 5.0 software (http://www.blast2go.org/).

### 4.6. Validation of Mature miRNAs and Corresponding Target Genes

To confirm the sequencing data, 13 miRNAs and 13 corresponding target genes were randomly selected for quantitative real-time RT-PCR (qRT-PCR). RNA used for qRT-PCR was the same as that used for library construction. For miRNA qRT-PCR, miRcute miRNA qPCR Detection Kit was used on a CFX96 Real-time System (BIO-RAD, Hercules, CA, USA) according to the manufacturer’s instructions. The forward primers of miRNAs were designed based on the mature miRNA sequence (Table S4) and the reverse primers were designed based on the adapter sequences, which were provided by the cDNA synthesis kit (TIANGEN, Beijing, China). For the qRT-PCR of target genes, specific primers (Table S5) were designed using the software Primer Premier 5.0 (PREMIER Biosoft Int., Palo Alto, CA, USA). The *B. napus Actin7* and *U6* snRNA of *B. napus* were used as the references for target genes and miRNAs, respectively. The relative expression level of each miRNA was calculated using the comparative method (2^−ΔΔCt^). Three biological replicates with three technical replicates for each miRNA were performed.

## Figures and Tables

**Table 1:** Statistical analysis of sequencing reads in three sRNA libraries in *B. napus*.

Samples	Raw_Reads	Low_Quality	containing’n’reads	Length < 18	Length > 30	Clean_Reads
CK	16,892,251	0	1164	575,989	2,514,501	13,800,597
T1	20,019,156	0	1413	799,829	5,296,926	13,920,988
T3	19,891,839	0	1417	1,122,546	2,965,574	15,802,302

**Table 2:** Known miRNA families and their transcript abundance in the three samples.

miRNA	Length	CK	T1	T3	Mature Sequence
miR1140	21	99.84	44.49	88.45	ACAGCCTAAACCAATCGGAGC
miR156a	21	25,154.03	16,041.64	13,758.44	TGACAGAAGAGAGTGAGCACA
miR156b	21	17,575.97	17,616.44	24,364.75	TTGACAGAAGATAGAGAGCAC
miR156d	20	25,634.20	16,450.91	14,216.79	TGACAGAAGAGAGTGAGCAC
miR159	21	283,188.49	271,657.99	306,215.83	TTTGGATTGAAGGGAGCTCTA
miR160a	21	2976.08	1975.18	3031.52	TGCCTGGCTCCCTGTATGCCA
miR161	21	57.05	53.38	152.78	TCAATGCACTGAAAGTGACTA
miR164a	21	2362.80	2411.14	2621.42	TGGAGAAGCAGGGCACGTGCA
miR164b	21	969.84	898.62	1045.35	TGGAGAAGCAGGGCACGTGCG
miR166a	21	100,777.77	105,636.37	92,513.67	TCGGACCAGGCTTCATTCCCC
miR166f	21	22,025.82	21,807.02	13,613.70	TCGGACCAGGCTTCATCCCCC
miR167a	22	13,753.66	10,694.43	9504.66	TGAAGCTGCCAGCATGATCTAA
miR167c	21	13,810.71	10,694.43	9617.24	TGAAGCTGCCAGCATGATCTA
miR167d	20	5357.89	3905.87	4615.63	TGAAGCTGCCAGCATGATCT
miR168a	21	13,929.56	14,404.56	14,787.71	TCGCTTGGTGCAGGTCGGGAA
miR169a	21	19.02	17.79	40.21	CAGCCAAGGATGACTTGCCGA
miR169g	22	9.51	0.00	0.00	TAGCCAAGGATGACTTGCCTGC
miR169m	21	19.02	8.90	24.12	TGAGCCAAAGATGACTTGCCG
miR169n	21	23.77	44.49	104.54	CAGCCAAGGATGACTTGCCGG
miR171a	21	456.40	631.70	321.65	TTGAGCCGTGCCAATATCACG
miR171f	21	1150.50	845.23	1085.56	TGATTGAGCCGCGCCAATATC
miR171g	22	1150.50	845.23	1085.56	TGATTGAGCCGCGCCAATATCT
miR172a	21	123.61	124.56	88.45	AGAATCTTGATGATGCTGCAT
miR172b	21	199.67	373.68	225.15	GGAATCTTGATGATGCTGCAT
miR172d	21	23.77	26.69	16.08	AGAATCTTGATGATGCTGCAG
miR2111a-3p	21	38.03	44.49	16.08	GTCCTCGGGATGCGGATTACC
miR2111a-5p	21	0.00	0.00	16.08	TAATCTGCATCCTGAGGTTTA
miR2111b-3p	21	85.57	35.59	64.33	ATCCTCGGGATACAGATTACC
miR2111c	21	4.75	0.00	0.00	TAATCTGCATCCTGGGGTTTA
miR390a	21	2700.34	1788.34	1648.44	AAGCTCAGGAGGGATAGCGCC
miR393	21	52.30	44.49	80.41	TCCAAAGGGATCGCATTGATC
miR394a	20	3342.14	3016.15	3337.09	TTGGCATTCTGTCCACCTCC
miR395a	21	16,382.69	21,735.84	27,243.49	CTGAAGTGTTTGGGGGAACTC
miR395d	21	6608.22	6317.01	5588.61	CTGAAGTGTTTGGGGGGACTC
miR397a	22	4.75	17.79	8.04	TCATTGAGTGCAGCGTTGATGT
miR399a	21	0.00	8.90	0.00	TGCCAAAGGAGATTTGCCCGG
miR403	21	26,927.32	24,342.72	26,310.71	TTAGATTCACGCACAAACTCG
miR6029	21	256.72	302.50	217.11	TGGGGTTGTGATTTCAGGCTT
miR6030	22	1093.45	818.54	522.68	TCCACCCATACCATACAGACCC
miR6031	24	1179.02	1574.80	868.45	AAGAGGTTCGGAGCGGTTTGAAGC
miR6034	21	19.02	26.69	88.45	TCTGATGTATATAGCTTTGGG
miR6035	21	61.80	160.15	88.45	TGGAGTAGAAAATGCAGTCGT
miR824	21	1911.16	1823.92	1286.59	TAGACCATTTGTGAGAAGGGA
miR860	21	4.75	8.90	32.16	TCAATACATTGGACTACATAT

**Table 3:** Predicted novel miRNA families and their transcript abundance identified in the three samples.

miRNA	Length	CK	T1	T3	Hairpin Energy (kcal moL^−1^)	Mature Sequence
Novel-001	21	128.4	160.1	72.4	−80.5	auaacuugguuuugcuccuac
Novel-002	21	12,845.6	12,011.2	14,924.4	−67.3	cggcucugauaccaauugaug
Novel-003	23	1625.9	2117.5	3264.7	−78.5	ugcucacggcucuuucugucagu
Novel-004	21	228.2	213.5	193.0	−73.8	ugccaaaggagaguugcccug
Novel-005	20	85.6	231.3	386.0	−61.2	uguguucucaggucaccccu
Novel-006	21	366.1	284.7	587.0	−51.2	uuggagcaucgagugaagagc
Novel-007	21	4278.7	3772.4	3393.4	−67.3	ucgauaaaccucugcauccag
Novel-008	24	2386.6	952.0	554.8	−72.9	gaugacgguaucucuccuacguag
Novel-009	22	23.8	17.8	217.1	−119.2	cagaagauguaugcuaaauugg
Novel-010	18	0.0	17.8	128.7	−49.4	gaggaaugaggaucauug
Novel-011	24	19,216.1	30,481.8	22,209.7	−69.5	agagauuuuuguuacuguuaacug
Novel-012	25	118.9	275.8	635.3	−57.7	gucaauugauggguaguaguucauu
Novel-013	24	2752.6	2811.5	1889.7	−60.5	agccuggcucugauaccaugaagu
Novel-014	21	294.8	266.9	241.2	−68.3	gacuuauaauaaucucaugaa
Novel-015	18	57.0	0.0	48.2	−69.1	cuuccaagaaaagcuuca
Novel-016	20	789.2	765.2	675.5	−75.9	ugaaugucuuucucuucauc
Novel-017	21	1141.0	1512.5	297.5	−76.1	uuguggaaccgugugaauacc
Novel-018	24	57.0	53.4	72.4	−55.4	ugaugugucaugucuagaaauccu
Novel-019	21	95.1	53.4	64.3	−95.6	uccccaguuuggauuguuugc
Novel-020	21	9555.8	7028.8	8202.0	−88	uaugugugcucacucucuauc
Novel-021	24	42.8	80.1	72.4	−56.1	acaggugguggaacaaauaugagu
Novel-022	21	713.1	409.3	289.5	−72.1	cgauauugguacgguugaauc
Novel-023	21	746.4	596.1	1101.6	−76.4	gauccucugaacacuucauug
Novel-024	24	142.6	329.2	176.9	−135.7	auuuuuagaacuucaauggguaga
Novel-025	21	171.1	160.1	257.3	−62.4	aucaugcgaucucuucggauu
Novel-026	21	26,375.8	21,495.6	24,670.3	−82.4	gcucucuagucuucugucauc
Novel-027	21	351.8	249.1	482.5	−75.1	guucccugaaacgcuucauug
Novel-028	20	8804.6	9164.1	9794.1	−80.6	uuggacugaagggagcuccu
Novel-029	21	309.0	302.5	442.3	−83.2	uuuugcguuucaacucggucc
Novel-030	21	13,311.5	10854.6	12,029.6	−70.1	cuugcauaucuuaggagcuuu
Novel-031	21	5191.5	2953.9	2830.5	−67.3	guucaauaaagcugugggaag
Novel-032	21	931.8	889.7	772.0	−80.4	uuggacugaagggaacucccu
Novel-033	24	156.9	222.4	241.2	−53.5	agcuaugguuuauguggacucagu
Novel-034	22	27,345.7	25,695.1	24,799.0	−72.4	gcucacugcucuuucugucaga
Novel-035	23	1606.9	1085.5	852.4	−74.3	gcucauucucguucugucauaac
Novel-036	21	1882.6	8719.2	10,807.3	−69.9	uguguucucaggucaccccug
Novel-037	21	1331.2	1245.6	1769.1	−88.6	gcuuacucucucucugucacc
Novel-038	23	156.9	302.5	233.2	−58.6	uucggaccaggcuucauucccca
Novel-039	23	608.5	1254.5	876.5	−80	guagguagacgcacuguuucuca
Novel-040	24	47.5	71.2	48.2	−85.1	ugaguuaucauuggucuuguguca
Novel-041	21	936.6	498.2	619.2	−71.8	gacuuauaaugaucucaugaa
Novel-042	21	185.4	329.2	209.1	−100.9	uugguuuaacuuggauuuuga
Novel-043	22	855.7	373.7	410.1	−70.6	cguacagaguagucaagcauga
Novel-044	21	256.7	462.7	257.3	−65.1	uguuuuguggguuucuaccga
Novel-045	21	2838.2	1868.4	3015.4	−95.5	cccgccuugcaucaacugaau
Novel-046	21	23,542.4	23,648.7	19,234.5	−83.2	uuggacugaagggagcucccu
Novel-047	24	584.8	898.6	619.2	−98.7	auauuccgauaagaacuucacucu
Novel-048	22	261.5	240.2	345.8	−79.4	uuucaucuuagagaauguuguc
Novel-049	22	551.5	284.7	241.2	−63.6	gcucucuauacuucugucaaua
Novel-050	23	2828.7	2615.8	2098.7	−53.2	uuggucacgugacuugugcuuua
Novel-051	24	5434.0	9635.7	6577.7	−57.3	aaacagcgguauucugacggacau
Novel-052	24	180.7	204.6	128.7	−49.4	aacuucggcuaagagauaguucuu
Novel-053	24	142.6	391.5	80.4	−49.7	aaauagaucuuuuugaaacaaauu
Novel-054	24	271.0	258.0	329.7	−60.1	auacagacucauauggaccaugug
Novel-055	21	375.6	293.6	514.6	−83.8	gcuuacucucucucugucacc
Novel-056	24	118.9	124.6	48.2	−51.2	aaagagcgacuauaguauaacuau
Novel-057	23	565.7	783.0	562.9	−67.3	agacuguaucuuauauuaugcua
Novel-058	21	266.2	160.1	144.7	−67.5	uugacagaagagagcgagcac
Novel-059	23	988.9	1067.7	1479.6	−88	ugcucaccucucuuucugucagu
Novel-060	21	1835.1	1708.3	1688.6	−85.5	uuuggauugaagggagcuccu
Novel-061	24	2572.0	4279.5	3473.8	−101.8	aggauuucauuuuccgucggaaug
Novel-062	24	14.3	177.9	64.3	−32.2	aaacgacguuguuuuauagcucuc
Novel-063	24	1373.9	2224.3	1897.7	−84.5	aggauuucauuuuccgucggaaug
Novel-064	24	3123.5	5035.8	4318.1	−114	aggauuucauuuuccgucggaaug
Novel-065	24	137.9	71.2	112.6	−26.9	aucuuaauugauugacaacucagc
Novel-066	24	589.5	702.9	418.1	−61.1	gauuaacccggaguucuuagaaug
Novel-067	24	285.2	427.1	217.1	−65.8	aggaugugguucuuagcggaaguu
Novel-068	24	347.1	240.2	249.3	−36.9	auaugaucugcacuuuugaacugu
Novel-069	20	7644.6	8399.0	8346.7	−77	ucccaaauguagacaaagca
Novel-070	24	3670.2	4341.8	3304.9	−59	agaauccgucucuuaacuuuuaac
Novel-071	21	698.9	1307.9	916.7	−76.8	uaagcugccagcaugaucuug
Novel-072	24	156.9	195.7	112.6	−87.5	aacgauuuuuguuacugauaacug
Novel-073	21	114.1	213.5	112.6	−63.2	uaugagaguauuauaagucac
Novel-074	21	389.8	231.3	225.2	−171.9	uaagacgagaaauugacaucc
Novel-075	24	247.2	480.4	659.4	−60.1	gucaacugauggugagagagaggu
Novel-076	21	332.8	756.3	1117.7	−86.8	uuguagaauuuugggaagggc
Novel-077	21	332.8	756.3	1166.0	−84.3	uuguagaauuuugggaagggc
Novel-078	21	318.5	444.9	450.3	−104.3	auuuucgauugguggauugug
Novel-079	22	556.2	320.3	249.3	−73.6	uuuugccuacuccucccauacc
Novel-080	20	8747.6	9173.0	9770.0	−80.7	uuggacugaagggagcuccu
Novel-081	21	38.0	80.1	160.8	−55.2	uggaugaugcuuggcucgaga
Novel-082	22	294.8	533.8	667.4	−78.9	ugguauugguaguaaugagugu
Novel-083	21	23,875.2	23,897.9	19,491.8	−87	uuggacugaagggagcucccu
Novel-084	22	76.1	106.8	209.1	−70.1	ucuugcuuaaauggguauucca
Novel-085	24	5196.2	15,258.7	5315.2	−32.2	agugcuauuuuugugacuuuugac
Novel-086	21	99,598.8	102,388.9	109,022.2	−79.3	uuuccaaauguagacaaagca
Novel-087	21	4350.0	4964.6	5693.1	−59.8	uuagaugaccaucaacaaaua
Novel-088	21	418.4	409.3	530.7	−65.5	ggacuguugucuggcucgagg
Novel-089	24	404.1	409.3	394.0	−79.6	caccgagguucguacggaggacgu
Novel-090	23	76.1	169.0	88.5	−68.6	gauacaugguuuguuagcugaua
Novel-091	20	42.8	106.8	201.0	−53.3	uguguucucaggucaccccu
Novel-092	21	8785.6	7687.2	6915.4	−64.9	ucgauaaaccucugcauccag
Novel-093	21	394.6	355.9	587.0	−63.1	uuugauuaggagcucauuguu
Novel-094	21	161.6	160.1	128.7	−67.8	ggacuguugucuggcucgagg
Novel-095	24	432.6	409.3	289.5	−54.7	aaaccgauucuauuagagcaugau
Novel-096	21	36,853.9	36,327.2	29,527.2	−97.3	uuggacugaagggagcucccu
Novel-097	22	451.6	524.9	377.9	−75.12	cuuaucucgaacgacuuucucg
Novel-098	24	52.3	275.8	297.5	−88.1	guuauuggaguuaauagagaaccg
Novel-099	21	841.5	6753.0	8177.9	−65.4	acagggaacaagcagagcaug
Novel-100	21	1359.7	783.0	675.5	−66	uucgcaggagagauagcgcca
Novel-101	24	1963.5	2188.7	2267.6	−123.2	ucuuaacuucggauaagagacggu
Novel-102	24	1621.2	1743.8	1616.3	−74.7	uauuauagagugaaccauuggagu
Novel-103	24	242.5	436.0	418.1	−36.3	aaugauaaggcgacuguaccagcu

## Supplementary Materials

The following are available online at http://www.mdpi.com/1422-0067/19/5/1431/s1, Table S1. Predicted target genes of miRNAs in this study. Table S2. Function descriptions of target genes predicted in this study. Table S3. Predicted target genes of differentially expressed miRNAs in this study. Table S4. Mature sequences of miRNAs used for qRT-PCR to validate sRNA sequencing data. Table S5. the primers used for qRT-PCR of target genes.
